# Migraine and the risk of glaucoma: a systematic review and meta-analysis

**DOI:** 10.3389/fneur.2026.1890818

**Published:** 2026-09-23

**Authors:** Sibiao Lu, Xiaojing Ye, Sitong Chen, Jisheng Zhang, Tianyi Sun, Xianming Lin

**Affiliations:** 1 The Third Clinical Medical College, Zhejiang Chinese Medical University, Hangzhou, China; 2 The Fourth Clinical Medical College, Zhejiang Chinese Medical University, Hangzhou, China; 3 The Third Affiliated Hospital, Zhejiang Chinese Medical University, Hangzhou, China

**Keywords:** glaucoma, meta-analysis, migraine, risk, systematic review

## Abstract

**Background:**

Migraine and glaucoma are prevalent and burdensome conditions worldwide, yet the evidence linking them remains inconsistent. This systematic review and meta-analysis aimed to synthesize available evidence on their association and investigate sources of heterogeneity.

**Methods:**

We comprehensively searched PubMed, Embase, and the Cochrane Library from inception to April 8, 2026, for cohort, case–control, and cross-sectional studies examining the migraine-glaucoma link. Pooled odds ratios (ORs) with 95% confidence intervals (CIs) were calculated using random-effects models. Heterogeneity was explored via subgroup, sensitivity, and meta-regression analyses. Study quality was assessed using the Newcastle-Ottawa Scale and the Agency for Healthcare Research and Quality checklist. Publication bias was evaluated via funnel plots, Egger’s test, and trim-and-fill analysis.

**Results:**

Nineteen studies involving 3,625,071 participants were incorporated. The pooled analysis demonstrated a significant positive association between migraine and glaucoma risk (OR = 1.28, 95% CI: 1.20–1.37), with moderate heterogeneity (I^2^ = 46.2%). Subgroup analyses indicated stronger associations in studies from America, clinic-based settings, those using self-reported migraine diagnosis and those with higher female participant proportions (≥60%). Egger’s test suggested potential publication bias. Following trim-and-fill adjustment (imputing eight potentially missing studies), the pooled estimate remained significant though slightly attenuated (OR = 1.23, 95% CI: 1.13–1.34).

**Conclusion:**

This meta-analysis suggests migraine is associated with a significantly increased risk of glaucoma. While the association persisted directionally after adjusting for potential publication bias, its magnitude varied by data source, diagnostic method, and population characteristics. Further high-quality prospective studies employing standardized criteria are needed to elucidate causality and underlying mechanisms.

**Systematic review registration:**

https://www.crd.york.ac.uk/PROSPERO/view/CRD420261367870, CRD420261367870.

## 1 Introduction

Migraine and glaucoma are among the most prevalent and burdensome public health conditions worldwide. Migraine, a complex neurological disorder, ranks as the second most disabling neurological condition globally, affecting approximately 14.1% of the population and serving as one of the most common forms of headache disorder (1). Beyond its hallmark symptom of recurrent, severe head pain, migraine significantly impacts daily functioning and social participation, contributing to profound disability, reduced workforce productivity, and a notable socioeconomic burden (2).

Conversely, glaucoma is a leading cause of irreversible blindness. It encompasses a heterogeneous group of progressive optic neuropathies with a pooled prevalence of 3.54% among individuals aged 40–80 years (3). Elevated intraocular pressure (IOP) remains the primary modifiable risk factor; however, conditions like normal-tension glaucoma, where optic nerve damage occurs despite normal IOP, highlight the multifactorial and not yet fully understood pathogenesis of the disease. As glaucoma often remains asymptomatic until advanced stages, it frequently evades early detection, necessitating lifelong monitoring and treatment. This incurs a substantial economic burden and significantly diminishes patients’ quality of life. Various pathophysiological mechanisms have been proposed to explain a potential link between migraine and glaucoma, including neuroinflammation (4, 5), vascular dysregulation, autonomic nervous system imbalance, and endothelial dysfunction (6). However, the precise nature and extent of this association require further elucidation (7).

Current observational studies investigating the association between migraine and glaucoma have produced inconsistent findings (8). Some studies report a significant positive association (9), while others find null (10) or even inverse relationships. These discrepancies may stem from several methodological limitations inherent to individual investigations, such as geographic variability, differences in sample sizes, study design variations, inadequate control for confounding variables, and inconsistencies in the definitions of migraine and glaucoma. As a result, these factors contribute to substantial heterogeneity. To date, we lack a comprehensive systematic review and meta-analysis that quantitatively synthesizes the available evidence on this association, highlighting a critical gap in the literature that this study aims to address.

Given the substantial impact of both migraine and glaucoma on individual quality of life and societal productivity, understanding their association carries significant clinical and public health implications. Such insights have the potential to inform evidence-based risk stratification, guide disease monitoring practices, advance mechanistic understanding, and support the development of targeted screening strategies for high-risk individuals. Therefore, this systematic review and meta-analysis aims to thoroughly synthesize the available evidence on the relationship between migraine and glaucoma risk, provide a robust pooled quantitative estimate of this association, and systematically investigate potential sources of heterogeneity. By treating migraine as the primary exposure of interest, this study seeks to quantify its relationship with glaucoma risk and explore—through pre-specified subgroup analyses—whether factors such as geographic region, study design, diagnostic methodology, and population characteristics moderate the observed association.

## 2 Methods

This systematic review and meta-analysis was conducted in accordance with the Preferred Reporting Items for Systematic Reviews and Meta-Analyses (PRISMA) 2020 statement (11). The study protocol was prospectively registered in the International Prospective Register of Systematic Reviews (PROSPERO; registration number: CRD420261367870).

### 2.1 Search strategy

A comprehensive literature search was performed across three electronic databases (PubMed, Embase, and the Cochrane Library) from their inception until April 8, 2026. The search strategy incorporated both medical subject headings (MeSH) and relevant keywords. Key search terms included “migraine disorders,” “glaucoma,” and “risk” (Supplementary Tables 1–3 for full details).

### 2.2 Study selection

Inclusion criteria: Exposure: diagnosis of migraine; Control: healthy population; Outcome: the risk of glaucoma and provided adequate data to calculate odds ratios (ORs) with 95% confidence intervals (CIs). Study design: observational studies, including cohort studies, case–control studies, and cross-sectional studies.

Exclusions consisted of conference abstracts, study protocols, and duplicate publications. When multiple publications originated from the same cohort, the study with the longest follow-up duration or the most comprehensive dataset was selected.

Two reviewers (Sibiao, Lu and Xiaojing, Ye) independently screened all retrieved records based on predefined inclusion and exclusion criteria. Any discrepancies were resolved through discussion with a third reviewer (Tianyi, Sun).

### 2.3 Data extraction

Extracted information included the first author’s name, publication year, study location, study design, participant source, method of migraine diagnosis, sex, age, sample size, and adjusted confounders. Two reviewers (Sitong, Chen and Tianyi, Sun) independently extracted relevant data, with disagreements resolved through consultation with a third reviewer (Jisheng, Zhang).

### 2.4 Quality assessment

Methodological quality of the included studies was evaluated using the Newcastle-Ottawa Scale (NOS) (12). The NOS score ranges from 0 to 9. Studies with scores > 7 were considered to be of high quality, those with scores of 4–6 were considered to be of moderate quality, and those with scores < 3 were considered to be of low quality. For cross-sectional studies, methodological quality was assessed using the 11-item checklist recommended by the Agency for Healthcare Research and Quality (AHRQ) (13). Each item was scored as 1 point if clearly satisfied and 0 points if not satisfied, unclear, or not reported, yielding a total score ranging from 0 to 11. Studies with scores ≥ 8 were considered to be of high quality, those with scores of 4–7 were considered to be of moderate quality, and those with scores ≤ 3 were considered to be of low quality.

### 2.5 Statistical analysis

The ORs and 95% CIs were extracted from each study to assess the association between migraine and glaucoma risk. For studies reporting RRs, we converted RRs to ORs using the baseline risk in the non-exposed/control group according to the inverse form of the method (14, 15):

$$\text{OR}=\frac{\mathrm{RR}(1-P_{0})}{1-\mathrm{RR}P_{0}}$$

Where $P_{0}$ denotes the event risk in the reference group. For studies reporting HRs, HRs were converted to approximate ORs at the reported follow-up time point under the proportional hazards assumption (16). The event risk in the exposed group was estimated as:

$$P_{1}(t)=1-{\left\{1-P_{0}(t)\right\}}^{\mathrm{HR}}$$

And subsequently converted to OR using

$$\text{OR}=\frac{P_{1}(t)/(1-P_{1}(t))}{P_{0}(t)/(1-P_{0}(t))}$$

If only unadjusted data were reported, crude ORs were calculated or extracted and included in the primary analysis, with the adjustment status recorded and evaluated in subgroup analyses. Considering the anticipated clinical and methodological heterogeneity across included studies, a random-effects model was used as the primary analytical approach to estimate the average effect across a distribution of true effects (17, 18). Statistical heterogeneity was assessed using I^2^ (19), τ^2^ and Cochran’s Q test. Sensitivity analysis was conducted by omitting one study at a time to test the robustness of the pooled results (20). Publication bias was assessed visually through funnel plots and statistically via Egger’s regression test (21). Subgroup analyses were performed based on region, study design, data source type, migraine diagnostic method, type of effect measure and proportion of female participants to explore potential sources of heterogeneity. Meta-regression was further employed to examine whether these variables influenced the overall results. All analyses were performed using Stata statistical software (version 14.0; StataCorp, College Station, Texas, United States).

## 3 Results

### 3.1 Study selection

The initial search of the three major electronic databases identified 453 records. After removal of duplicates, 363 records remained for title and abstract screening. Among these, 329 records were excluded based on their titles and abstracts. After full-text review, an additional 21 studies were excluded: 15 studies (7, 22–35) did not evaluate migraine as an exposure, 3 studies (36–38) did not report glaucoma as an outcome, 2 studies (39, 40) were derived from the same cohort, and 1 study (41) was unavailable in full text. Furthermore, 6 additional studies were identified through reference screening and manual searching. Ultimately, 19 studies were included in the final meta-analysis (Figure 1).

**Figure 1:**
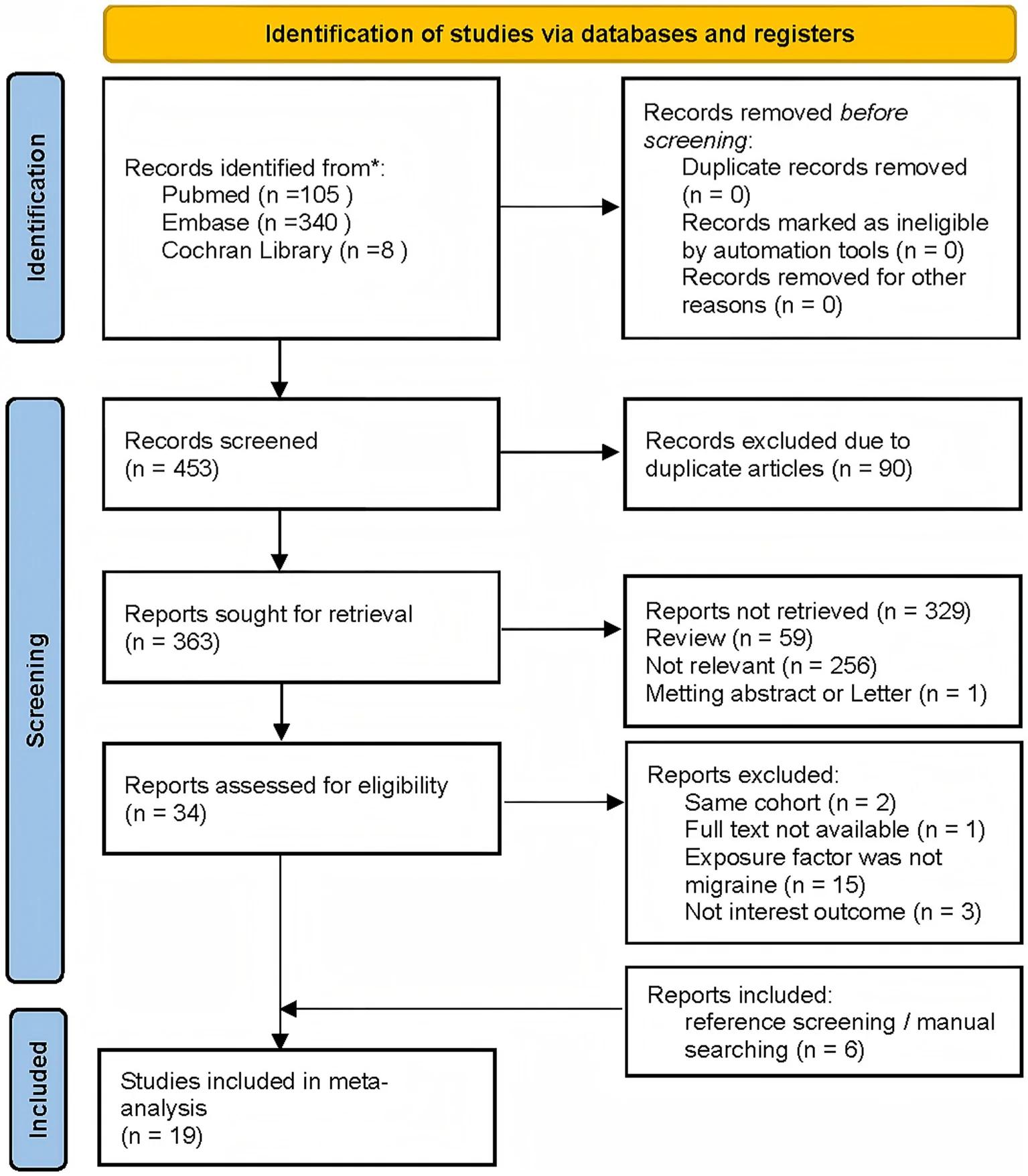
Study flowchart depicting the methodology used in selecting articles for inclusion in the study.

### 3.2 Study characteristics

Nineteen studies (6, 9, 10, 42–57), encompassing a total of 3,625,071 participants with a mean age of 55.0 years, were included (Table 1). Geographically, the studies were distributed as follows: 9 in Asia, 6 in America, 3 in Europe, and 1 in Oceania. By design, there were 9 cohort studies, 8 case–control studies, and 2 cross-sectional studies. Regarding data sources, 10 studies were based on clinical investigations, while 9 utilized data from electronic databases. Migraine ascertainment relied on physician diagnosis in 13 studies and on self-report in 6 studies. All the included studies employed multivariable models to adjust for potential confounders. The participant pool comprised both males and females in 16 studies; sex distribution was not reported in 2 studies, and 1 study included only female participants.

**Table 1:** Characteristics of the included studies.

Study	Year	Country	Study design	Sample size	Female%	Age, years	Migraine ascertainment	Glaucoma assessment	Multivariable adjustment
Lee et al. (42)	2026	Korea	Retrospective cohort study	Total: 168 Case: 44 Control: 124	60.4%	Case: 60.7(9.9) Control: 61.1(14.4) (mean)	Self-report	Clinical diagnosis based on optic disc/RNFL and visual field criteria	Percentage reduction in IOP disc hemorrhage
Bar et al. (9)	2026	Southern Israel	Retrospective cohort study	Total: 83758 Case: 30733 Control: 53025	74.5%	NA	Medical records	ICD-9 codes 365.0–365.9	Age, sex, Jewish ethnicity, education, family status, socioeconomic index, comorbidities, and age at first ophthalmology visit
Kim et al. (43)	2025	Korea	Retrospective cohort study	Total: 246889 Case: 41148 Control: 205741	70.7%	NA	Medical records	KCD codes H401	Age, sex, income, residential area, CCI, and comorbidities including hypertension, diabetes mellitus (DM), hyperlipidemia, and ischemic stroke
Ohn et al. (44)	2023	Korea	Retrospective cohort study	Total: 2716562 Case: 87809 Control: 2628753	49.7%	Case: 56.46(10.91) Control: 54.14(10.4) (mean)	Medical records	ICD-10 code for POAG (H401)	Age, sex, lifestyle factors (smoking, drinking, and body mass index (BMI)), and comorbidities (diabetes, hypertension, and dyslipidemia).
Funk et al. (45)	2022	America	Case–control study	Total: 554 Case: 277 Control: 277	57.4%	Case: 69.5(10.5) Control: 68.9(10) (mean)	Medical records	ICD-9 code 365.12	Age, sex
Huang et al. (6)	2019	China	Retrospective cohort study	Total: 86415 Case: 17283 Control: 69132	73.2%	Case: 45.3(14.9) Control: 45.3(14.9) (mean)	Medical records	ICD-9-CM codes 365.11–365.12	Age, sex, hyperlipidemia, ischemic heart disease, hypertension, peripheral vascular disease, cerebrovascular disease, and diabetes mellitus simultaneously
Chen et al. (10)	2016	China	Retrospective cohort study	Total: 88029 Case: 17606 Control: 70423	73.5%	Case: 45.1(14.8) Control: 45.1(14.9) (mean)	Medical records	ICD-9-CM code 365.1x; ICD-9-CM code 365.2x	Age, sex, and comorbidities of diabetes, hypertension, hyperlipidemia, and coronary artery disease
Furlanetto et al. (46)	2014	America	Prospective cohort study	Total: 127 Case: 127 Control: NA	58.0%	Case: 64.7(10.9) Control: NA (mean)	Medical records	Clinical diagnosis of LTG based on IOP optic disc and visual field criteria	Sex, treatment randomization, Raynaud phenomenon, migraine, baseline neuroretina rim width, systemic β-blocker use, systolic blood pressure, and mean arterial ocular perfusion pressure
Lin et al. (47)	2010	China	Case–control study	Total: 306692 Case: 76673 Control: 230019	47.8%	Case: 62.7(13.1) Control: 62.7(13.1) (mean)	Medical records	ICD-9 codes 365.1–365.11	Age, sex, monthly income and level of urbanization of the community in which the patient resided
Lin et al. (48)	2010	China	Retrospective cohort study	Total: 2313 Case: 97 Control: 2216	54.0%	Total: 69.4(6.9) (mean)	Medical records	ICD-9 codes 365.1–365.11	Age, sex
Welinder et al. (49)	2009	Denmark	Case–control study	Total: 65901 Case: 5991 Control: 59910	58.4%	Case: 70.4(60.8–78.4) Control: 70.3(60.8–78.4)(median)	Medical records	Prescription-based glaucoma definition	Age, sex, thyroid disease, migraine, autoimmune disorders, cardiovascular events, hypertension, alcohol-related disorders, ACE inhibitors, oral beta-adrenoceptor blockers and statins
Motsko et al. (50)	2008	Portugal	Case–control study	Total: 18912 Case: 4728 Control: 14184	61.7%	Case: 73.6(7.6) Control: 73.5(7.6) (mean)	Medical records	ICD-9-CM code 365.1	Age, sex
Girkin et al. (51)	2004	America	Nested case–control study	Total: 6487 Case: 590 Control: 5897	0%	Case: 69 Control: 69 (mean)	Medical records	ICD-9 code 365.1	Age, sex
Landers et al. (52)	2002	Australia	Case–control study	Total: 739 Case: 438 Control: 301	59.3%	Case: 59(13) Control: 51(12.6) (mean)	Medical records	Clinical diagnosis of POAG based on IOP optic disc and visual field criteria	Age, sex, IOP,systemic hypertension, diabetes, Raynaud’s phenomenon, myopia family history and referral source
Drance et al. (53)	2001	America	Prospective cohort study	Total: 160 Case: 52 Control: 108	61.9%	Case: 63.66(9.86) Control: 63.66(9.86) (mean)	Self-report	Clinical diagnosis of NTG based on optic disc and visual field criteria	Age, sex, migraine, baseline hemorrhages, baseline median intraocular pressure, baseline mean deviation and hypertension
Cursiefen et al. (54)	2000	Germany	Cross-sectional case–control study	Total: 229 Case: 75 Control: 154	44.34%	Case: 44(16) Control: 55(11) (mean)	Self-report	Clinical diagnosis of NPG/HPG based on IOP optic disc/RNFL and visual field criteria	Sex
Usui et al. (55)	1991	Japan	Cross-sectional case–control study	Total: 225 Case: 150 Control: 75	51.99%	Case: 58.5(13.4) Control: 57.6(14.2) (mean)	Self-report	Clinical diagnosis and subtype classification based on IOP	Age, sex
Phelps and Corbett (56)	1985	America	Case–control study	Total: 729 Case: 236 Control: 493	68.3%	NA	Self-report	Clinical classification of LTG and POAG	Age, sex
Morgan and Drance (57)	1975	Canada	Case–control study	Total: 182 Case: 91 Control: 91	NA	NA	Self-report	Clinical classification of COAG and OHT	Age, sex, education and income levels

ACE, Angiotensin-Converting Enzyme; BMI, Body Mass Index; CCI, Charlson Comorbidity Index; COAG, Chronic Open-Angle Glaucoma; DM, Diabetes Mellitus; HPG, High-Pressure Glaucoma; ICD, International Classification of Diseases Code; ICD-9-CM, International Classification of Diseases Ninth Revision Clinical Modification; IOP, Intraocular Pressure; KCD, Korean Standard Classification of Diseases; LTG, Low-Tension Glaucoma; NA, Not Available; NPG, Normal-Pressure Glaucoma; NTG, Normal-Tension Glaucoma; OAG, Open-Angle Glaucoma; OHT, Ocular Hypertension; POAG, Primary Open-Angle Glaucoma; PPG, Preperimetric Glaucoma; RNFL, Retinal Nerve Fiber Layer.

### 3.3 Risk of bias

Based on the NOS assessment, the quality scores of individual studies ranged from 6 to 8 (Supplementary Tables 4 for full details). According to the quality assessment results, a total of 13 studies were rated as high quality and 4 studies as moderate quality, with no studies classified as low quality. The main factors contributing to reduced scores included insufficient description of participant selection or control selection, inadequate control of potential confounding factors, and incomplete reporting of exposure and outcome assessment or follow-up completeness. Based on the AHRQ checklist, the two cross-sectional studies scored 4 and 5 points (Supplementary Tables 5 for full details), respectively, indicating that both were of moderate methodological quality.

### 3.4 Migraine and risk of glaucoma

All 19 studies contributed data to the primary analysis. The pooled analysis, conducted using a random-effects model, revealed a statistically significant association between migraine and glaucoma (OR = 1.28, 95% CI: 1.20–1.37), with moderate heterogeneity observed (Cochran’s Q = 33.44, *I*^2^ = 46.2%, *p* = 0.015) (Figure 2). Sensitivity analysis, performed by omitting one study at a time, confirmed the robustness of this pooled estimate (Supplementary Figure 1).

**Figure 2:**
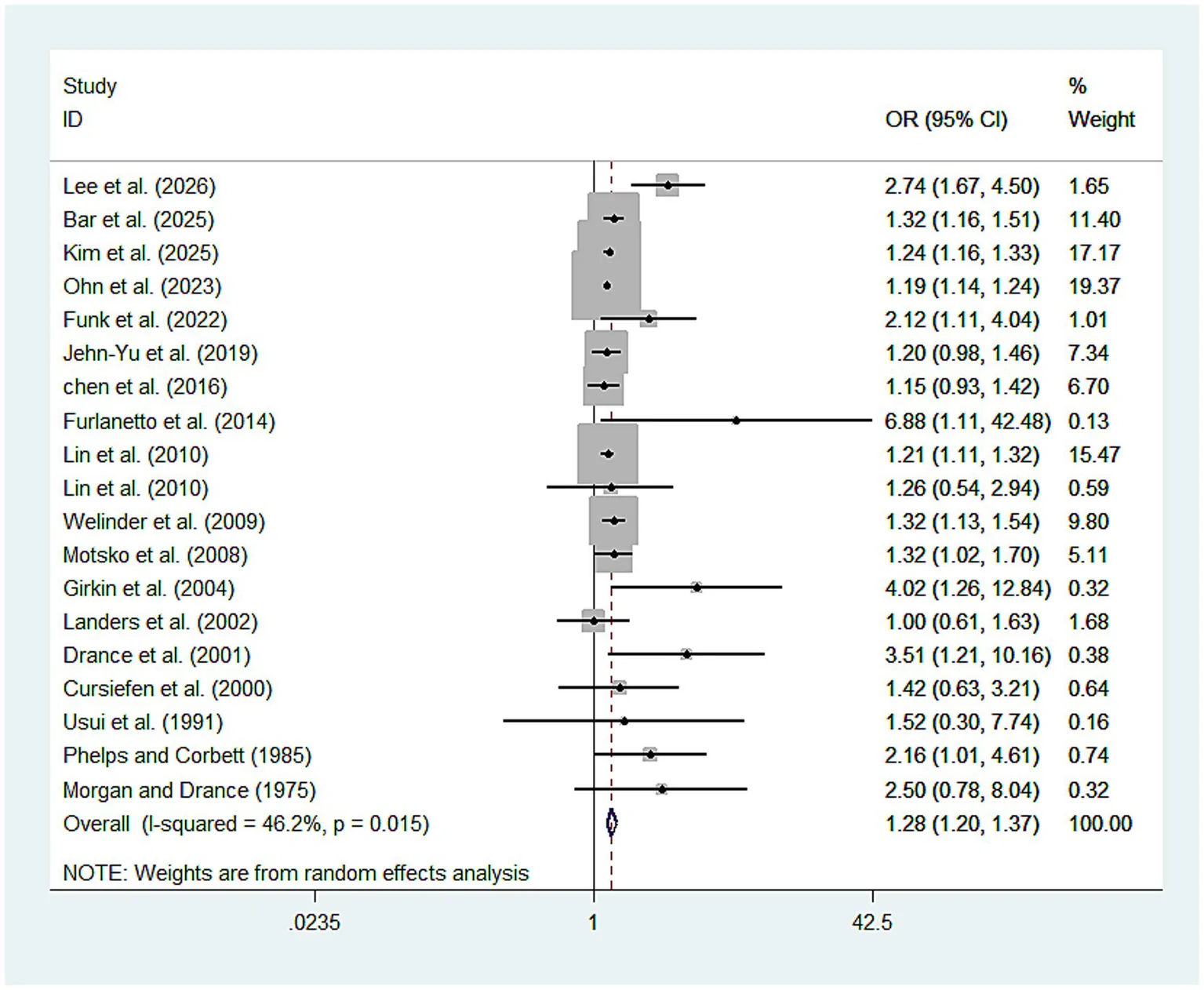
Forest plot of the association between migraine and glaucoma.

### 3.5 Subgroup analyses

Studies conducted in North America indicated a significantly elevated risk with no heterogeneity (OR = 2.62, 95% CI: 1.78–3.84, *I*^2^ = 0.0%, *p* = 0.767). Analyses by study design showed that cohort studies (OR = 1.27, 95% CI: 1.16–1.39, *I*^2^ = 61.4%, *p* = 0.008), case–control studies (OR = 1.33, 95% CI: 1.16–1.54, *I*^2^ = 40.5%, *p* = 0.109) and cross-sectional studies (OR = 1.44, 95% CI: 0.69–2.99, *I*^2^ = 0.0%, *p* = 0.328) yielded similar results.

When stratified by data source, studies based on clinic-based studies demonstrated a significantly elevated odds ratio (OR = 2.08, 95% CI: 1.50–2.92, *I*^2^ = 37.6%, *p* = 0.108), whereas those based on databases showed a more modest but significant association (OR = 1.22, 95% CI: 1.18–1.25, *I*^2^ = 0.0%, *p* = 0.802). The method of migraine diagnosis also influenced the effect size: self-reported diagnosis was associated with a stronger effect (OR = 2.34, 95% CI: 1.69–3.25, *I*^2^ = 0.0%, *p* = 0.744) compared to physician diagnosis (OR = 1.23, 95% CI: 1.18–1.29, *I*^2^ = 23.0%, *p* = 0.211).

Furthermore, the association appeared stronger in studies with ≥60% female participants (OR = 1.33, 95% CI: 1.18–1.50, *I*^2^ = 58.0%, *p* = 0.020) than in those with <60% females (OR = 1.22, 95% CI: 1.14–1.30, *I*^2^ = 21.5%, *p* = 0.258) (Table 2). Meta-regression analysis identified data source (*β* = −0.47, 95% CI: −0.75 to −0.20, *p* = 0.002) and migraine diagnostic method (*β* = −0.65, 95% CI: −1.01 to −0.29, *p* = 0.002) as significant contributors to the observed heterogeneity.

**Table 2:** Subgroup analysis.

Subgroup	No. study	Test of association			Test of heterogeneity	
		OR	95% CI	*p* value	*p* value	*I* ^2^
Region						
Asia	9	1.232	1.165–1.303	<0.001	0.096	40.7%
Europe	3	1.322	1.160–1.507	<0.001	0.985	0.0
America	6	2.616	1.783–3.839	<0.001	0.767	0.0
Oceania	1	1.000	0.612–1.633	1.000	/	/
Study design						
Cohort	9	1.268	1.159–1.386	<0.001	0.008	61.4%
Case–control	8	1.333	1.158–1.536	<0.001	0.109	40.5%
Cross-sectional	2	1.440	0.694–2.985	0.328	0.942	0.0
Data source type						
Clinic trial-based	10	2.084	1.489–2.916	<0.001	0.108	37.6%
Database-based	9	1.215	1.178–1.253	<0.001	0.802	0.0
Diagnosis of migraine						
Self-reported	6	2.339	1.686–3.246	<0.001	0.744	0.0
Physician diagnosis	13	1.233	1.178–1.291	<0.001	0.211	23.0%
Effect value type						
Adjusted	12	1.253	1.177–1.334	<0.001	0.019	51.7%
Crude	7	1.933	1.382–2.703	<0.001	0.746	0.0
Female proportion						
≥60%	8	1.328	1.178–1.498	<0.001	0.020	58.0%
<60%	8	1.222	1.144–1.304	<0.001	0.258	21.5%

### 3.6 Publication Bias

Visual inspection of the funnel plot suggested asymmetry, indicating potential publication bias in the association between migraine and glaucoma risk (Figure 3). This was statistically confirmed by Egger’s regression test (*p* < 0.001). To address this asymmetry, the trim-and-fill method was applied. After three iterations, eight potentially missing studies were imputed, adjusting the total number of studies to 27. Following this correction, no obvious publication bias remained. The adjusted pooled effect estimate incorporating the imputed studies was OR = 1.23 (95% CI: 1.13–1.34).

**Figure 3:**
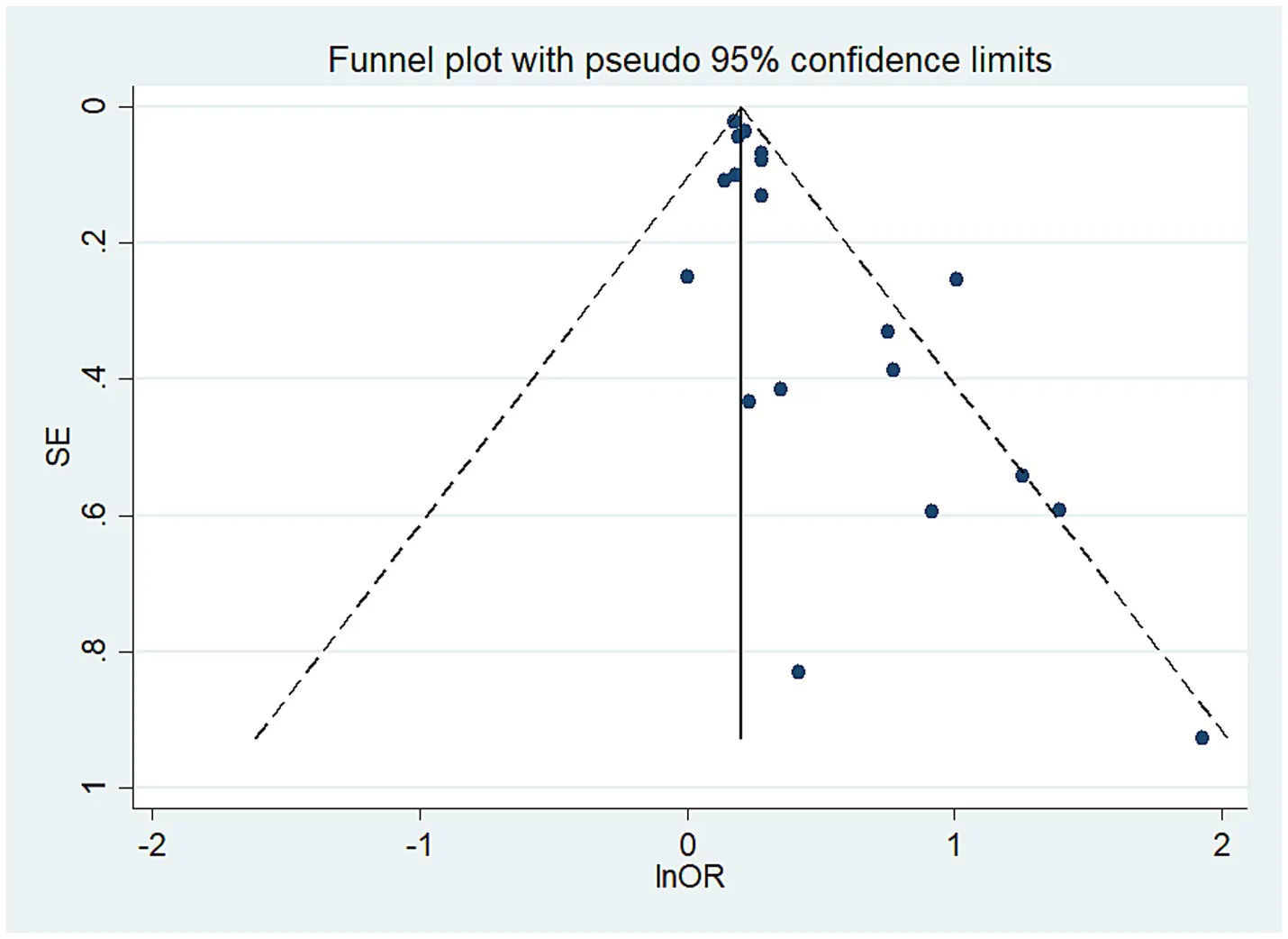
Publication bias of the association between migraine and glaucoma.

## 4 Discussion

### 4.1 Main finding

This meta-analysis demonstrates a statistically significant positive association between migraine and the risk of glaucoma. Individuals with migraine were found to have 28% higher odds of developing glaucoma compared to those without migraine. This pooled estimate remained robust in sensitivity analyses, supporting the reliability of the finding.

### 4.2 Comparison and interpretation with previous studies

Our findings are consistent with prior research, corroborating migraine as a significant risk factor for glaucoma (8). However, earlier meta-analyses were limited by a lack of rigorous heterogeneity investigation and did not include more recent observational evidence. The present study advances the field by synthesizing a more comprehensive and updated body of evidence with a substantially larger pooled sample size. Through detailed subgroup and meta-regression analyses, we systematically explored sources of heterogeneity, identifying both the data source and the method of migraine diagnosis as significant contributors.

Regarding diagnostic method, studies relying on self-reported migraine yielded a markedly higher pooled effect estimate (OR = 2.34) than those using physician diagnosis (OR = 1.23). This discrepancy is likely multifactorial. Self-report is susceptible to recall bias and subjective symptom interpretation, potentially leading to over-reporting among symptomatic or health-conscious individuals, which may inflate the effect size and compromise diagnostic specificity. In contrast, physician-based diagnosis, while more stringent, may result in the under-ascertainment of mild or atypical migraine presentations. Furthermore, patients who receive a formal clinical diagnosis are more likely to undergo structured evaluation and management, which could facilitate the incidental detection of early ocular changes and potentially attenuate the observed association with glaucoma. Further clinical and pathological observations are consistent with this proposed vascular mechanism. In a study of 51 patients with intracranial vascular malformations, 47% reported migraine-like headaches. This association was more evident in patients with occipital arteriovenous malformations, and headache improved in some patients after lesion eradication (58, 59). These findings suggest that local hemodynamic disturbances, perivascular nociceptive signaling, and trigeminovascular activation may contribute to migraine in susceptible individuals. In addition, vascular wall abnormalities have been observed in the superficial temporal and occipital arteries of patients undergoing migraine surgery, although the sample size was limited (60). A case report describing improvement of chronic migraine after ligation of a superficial temporal artery aneurysm further suggests that abnormal vascular pulsation may act as a local mechanical trigger (61). However, these observations are based mainly on small case series or case reports and should not be interpreted as evidence of a universal structural vascular lesion in migraine.

With respect to data source, clinic-based studies (OR = 2.08) showed a stronger association than database-derived studies (OR = 1.22). This difference may be explained by methodological variation and selection bias. Clinic-based studies typically employ rigorous, standardized diagnostic protocols, and patient populations in specialty settings may present with more severe symptoms, greater comorbidity clustering, or higher healthcare utilization, which may introduce Berkson’s bias (62) and overrepresent the co-occurrence of migraine and glaucoma. In addition, migraine patients in clinical settings may undergo more frequent ophthalmic examinations or medical evaluations, thereby increasing the probability of glaucoma detection and consequently introducing detection or surveillance bias. Accordingly, the higher pooled estimate among clinic-based studies may, at least in part, reflect differences in patient selection, diagnostic intensity, healthcare-seeking behavior, and residual confounding rather than solely a stronger underlying biological association. Database studies, while offering advantages in sample size and generalizability, are inherently susceptible to diagnostic coding inaccuracies, potential false-negative classifications, and variability in coding practices, which may introduce non-differential misclassification and attenuate effect estimates. Furthermore, the relatively high heterogeneity observed in the cohort-study subgroup may reflect the fact that this category encompasses both prospective and retrospective cohort studies. These studies differ in design, including follow-up structure, the ascertainment of exposures and outcomes, reliance on administrative coding, and adjustment for potential confounders, all of which may contribute to residual between-study variability.

Subgroup analyses also revealed that the association was more pronounced in studies with a higher proportion of female participants (≥60%). This is biologically plausible given the well-established female preponderance in migraine (63), largely attributed to hormonal influences. Estrogen modulates ocular vascular tone and intraocular pressure homeostasis, suggesting a potential shared pathway influencing both conditions. Interestingly, while women are more susceptible to migraine, epidemiological data indicate a higher risk of primary open-angle glaucoma (POAG) in men (3). This paradox may be partially explained by emerging evidence for a neuroprotective role of estrogen in the optic nerve (64), which could confer relative protection against glaucomatous damage in women.

The stronger association observed in studies from America should be interpreted cautiously because this subgroup included only six studies. Although statistical heterogeneity was low in this subgroup, the small number of studies limits the reliability of the *I*^2^ estimate and does not exclude clinically or methodologically meaningful differences. Several factors may have contributed to the higher pooled estimate, including differences in study design, glaucoma definitions, migraine ascertainment, control selection, and covariate adjustment. In particular, some American studies focused on low-tension or normal-tension glaucoma/optic neuropathy-related outcomes and used self-reported migraine or medical-record-based ascertainment, whereas many Asian studies were large retrospective database studies using administrative diagnostic codes. Therefore, the regional subgroup finding should be regarded as exploratory and hypothesis-generating rather than evidence of a definitive geographic effect. From a clinical perspective, migraine with aura is associated with an increased risk of ischemic stroke. New-onset, persistent, atypical headaches, or headaches accompanied by focal neurological signs, should prompt evaluation for secondary vascular causes (65). Pattern glare, pupillary abnormalities, and visual-field changes may also influence neurovascular coupling through cortical hyperexcitability (66). However, current evidence is insufficient to determine whether vascular abnormalities precede migraine or glaucoma, or whether a causal relationship exists. Therefore, prospective studies are needed to integrate standardized migraine and glaucoma phenotyping with vascular imaging, endothelial biomarkers, and autonomic biomarkers to further clarify the clinical significance and pathophysiological mechanisms underlying this association.

### 4.3 Possible biological mechanisms

The observed positive association between migraine and glaucoma risk may be explained by several shared pathophysiological mechanisms. Although the observational nature of the included studies precludes causal inference, converging evidence points to common pathways involving vascular dysregulation, neuroinflammation with oxidative stress, and genetic susceptibility.

Both migraine and glaucoma—particularly normal-tension glaucoma—share key pathophysiological features: systemic vascular dysregulation (28) and autonomic dysfunction (67, 68). During migraine attacks, cortical spreading depression triggers vasospasm extending to the ocular circulation, reducing blood flow velocity in the central retinal and posterior ciliary arteries and compromising perfusion at the optic nerve head (69). Elevated endothelin-1 (ET-1) during migraine not only constricts optic nerve vessels (70, 71) but may directly induce retinal ganglion cell apoptosis (72), linking vascular and neurotoxic mechanisms.

Activation of the trigeminovascular system releases calcitonin gene-related peptide (CGRP), whose receptors are expressed in the retinal nerve fiber layer (28, 67), suggesting that migraine-related signaling may disturb the ocular neurovascular environment. Both conditions involve chronic neuroinflammation and oxidative stress: glaucoma is associated with elevated pro-inflammatory cytokines and microgial activation (73), while recurrent ischemia–reperfusion during migraine attacks generates reactive oxygen species that may synergize with mitochondrial dysfunction in glaucoma (74, 75).

Shared genetic factors may also contribute. The C677T polymorphism in the MTHFR gene elevates homocysteine, which in migraine lowers the threshold for cortical spreading depression via NMDA receptor-mediated excitotoxicity (76), while in glaucoma it promotes endothelial toxicity and extracellular matrix remodeling (77). These convergent vascular, inflammatory, and genetic pathways may represent key mechanisms linking migraine to glaucomatous injury independent of intraocular pressure. It should be noted that this mechanistic framework is presented to generate research hypotheses rather than to establish causality, which requires further experimental validation.

### 4.4 Strengths and limitations

This meta-analysis has several strengths. It was conducted according to PRISMA guidelines, with a prospectively registered protocol. The large cumulative sample size, broad geographic coverage, and inclusion of diverse study designs enhance the generalizability of the findings. Extensive subgroup analyses and meta-regression were performed to explore sources of heterogeneity, yielding novel insights into the association.

Several limitations should be acknowledged. First, Egger’s test indicated publication bias. Trim-and-fill imputation suggested that approximately eight negative or weakly positive studies might be missing, reducing the pooled OR from 1.29 to 1.23, suggesting that the original estimate may have been slightly inflated. However, the direction of the association remained unchanged, supporting the robustness of the main finding. The magnitude of the association between migraine and glaucoma should be interpreted with caution, as the true effect may be closer to the adjusted estimate obtained under the trim-and-fill method (OR = 1.23). The funnel plot asymmetry may reflect publication bias, small-study effects, or methodological heterogeneity, rather than publication bias alone. Second, most included studies were observational, precluding causal inference. Heterogeneity in diagnostic criteria—especially between self-reported and physician-diagnosed migraine—was a notable source of between-study variation. Third, although most studies reported adjusted estimates, residual confounding from unmeasured variables (78) (e.g., vascular risk factors, medication use, lifestyle, genetic predisposition) cannot be ruled out. Pharmacological confounding is particularly complex: triptans may increase ocular ischemia risk (79), whereas beta-blockers (80) could improve perfusion; calcium channel blockers (81) have shown ambiguous effects. Fourth, geographic representation was uneven, with limited data from Oceania and some other regions, which may affect the global applicability of the findings. Fifth, most studies did not stratify by migraine or glaucoma subtypes, preventing subtype-specific analyses that could have pathophysiological and clinical relevance. Sixth, follow-up durations were generally insufficient to assess long-term risk or temporal dynamics of the association. Eighth, this study included two cross-sectional studies of moderate quality. Although these studies met the inclusion criteria, their low-to-moderate methodological quality may introduce bias related to participant selection, exposure/outcome ascertainment, and incomplete adjustment for confounders. Their findings should therefore be interpreted with caution.

### 5 Conclusion

Based on the available evidence, the present meta-analysis demonstrates that migraine is significantly associated with an increased risk of glaucoma (OR = 1.28). However, the magnitude of this association was substantially influenced by geographic region, migraine diagnostic method, and data source, and the presence of publication bias or small-study effects may have inflated the current pooled estimate, indicating that the magnitude of the association is modest.

Accordingly, the findings of this meta-analysis should be interpreted with appropriate caution. Future high-quality prospective studies employing standardized diagnostic criteria and rigorous methodological frameworks are warranted to further substantiate the causal nature of this relationship and to elucidate the underlying pathophysiological mechanisms linking migraine to glaucoma risk.

## Data Availability

The original contributions presented in the study are included in the article/Supplementary material, further inquiries can be directed to the corresponding author/s.
