# Supplementary material for: Migraine and the risk of glaucoma: a systematic review and meta-analysis
Source: Front Neurol. 2026 Sep 23;17:1890818. doi: 10.3389/fneur.2026.1890818 (PMC13642578; doi:10.3389/fneur.2026.1890818)
Supplement: Supplementary file 1 [file Supplementary_file_1.DOCX]

Supplementary Material

**Supplementary Tables 1 Search strategy of Pubmed**

**Supplementary Tables 2 Search strategy of EMBASE**

**Supplementary Tables 3 Search strategy of Cochrane Library**

**Supplementary Tables 4 The quality assessment of cohort and case-control studies**

**Supplementary Tables 5 The quality assessment of Cross-sectional studies**

**Supplementary Tables 6 Meta-Regression of Heterogeneity Factors in Migraine and All-Cause Mortality**

**Supplementary Tables 7 Original data extraction and transformation**

**Supplementary Figure 1 Sensitivity analysis of the association between migraine and glaucoma**

**Supplementary Tables 1 Search strategy of Pubmed**

| No. | Content | Result |
| --- | --- | --- |
| #1 | Search: "Migraine Disorders"[Mesh] Sort by: Most Recent | 35,087 |
| #2 | Search: Migrain*[Title/Abstract] Sort by: Most Recent | 49,711 |
| #3 | #1 OR #2: Search: ("Migraine Disorders"[Mesh]) OR (Migrain*[Title/Abstract]) Sort by: Most Recent | 53,943 |
| #4 | Search: "Glaucoma"[Mesh] Sort by: Most Recent | 64,843 |
| #5 | Search: Glaucoma*[Title/Abstract] Sort by: Most Recent | 81,582 |
| #6 | #4 OR #5: Search: ("Glaucoma"[Mesh]) OR (Glaucoma*[Title/Abstract]) Sort by: Most Recent | 92,293 |
| #7 | Search: "Risk"[Mesh] Sort by: Most Recent | 1,529,018 |
| #8 | Search: risk*[Title/Abstract] Sort by: Most Recent | 3,638,333 |
| #9 | #7 OR #8: Search: ("Risk"[Mesh]) OR (risk*[Title/Abstract]) Sort by: Most Recent | 4,148,849 |
| #10 | #3 AND #6 AND #9:  Search: ((("Migraine Disorders"[Mesh]) OR (Migrain*[Title/Abstract])) AND (("Glaucoma"[Mesh]) OR (Glaucoma*[Title/Abstract]))) AND (("Risk"[Mesh]) OR (risk*[Title/Abstract])) Sort by: Most Recent | 105 |

**Supplementary Tables 2 Search strategy of EMBASE**

| No. | Content | Result |
| --- | --- | --- |
| #1 | 'migraine' | 106,174 |
| #2 | 'migrain*':ab,ti | 80,923 |
| #3 | #1 OR #2 | 107,110 |
| #4 | 'glaucoma' | 140,209 |
| #5 | 'glaucoma*':ab,ti | 105,008 |
| #6 | #4 OR #5 | 141,177 |
| #7 | 'risk' | 6,175,221 |
| #8 | 'risk*':ab,ti | 5,329,639 |
| #9 | #7 OR #8 | 6,397,937 |
| #10 | #3 AND #6 AND #9 | 340 |

**Supplementary Tables 3 Search strategy of Cochrane Library**

| No. | Content | Result |
| --- | --- | --- |
| #1 | MeSH descriptor: [Migraine] this term only | 11,060 |
| #2 | (migrain*):ab,ti,kw | 10,966 |
| #3 | #1 or #2 | 11,216 |
| #4 | MeSH descriptor: [Glaucoma] this term only | 9,724 |
| #5 | (glaucoma*):ab,ti,kw | 9,504 |
| #6 | #4 or #5 | 9,827 |
| #7 | MeSH descriptor: [Risk] this term only | 345,313 |
| #8 | (risk*):ab,ti,kw | 351,628 |
| #9 | #7 or #8 | 359,368 |
| #10 | #3 and #6 and #9 | 20(trial=8) |

**Supplementary Tables 4 The quality assessment of cohort and case-control studies**

| Study | Year | Selection(4) | Comparability(2) | Outcome(3) | Total |
| --- | --- | --- | --- | --- | --- |
| Case-control studies (n=8) | | | | | |
| Funk et al. | 2022 | *** | * | ** | 6 |
| Lin et al. | 2010 | **** | * | *** | 8 |
| Welinder et al. | 2009 | **** | ** | ** | 8 |
| Motsko et al. | 2008 | *** | * | *** | 7 |
| Girkin et al. | 2004 | ** | * | *** | 6 |
| Landers et al. | 2002 | * * | ** | ** | 6 |
| Phelps and Corbett | 1985 | ** | * | *** | 6 |
| Morgan and Drance | 1975 | ** | ** | *** | 7 |
| Cohort studies (n=9) | | | | | |
| Lee et al. | 2026 | *** | ** | ** | 7 |
| Bar et al. | 2025 | *** | ** | ** | 7 |
| Kim et al. | 2025 | **** | ** | ** | 8 |
| Ohn et al. | 2023 | **** | ** | ** | 8 |
| Jehn-Yu et al. | 2019 | **** | ** | ** | 8 |
| chen et al. | 2016 | **** | ** | ** | 8 |
| Furlanetto et al. | 2014 | *** | ** | ** | 7 |
| Lin et al. | 2010 | **** | ** | ** | 8 |
| Drance et al. | 2001 | *** | ** | ** | 7 |

The NOS scale was used to evaluate the quality of the cohort and case-control study

**Supplementary Tables 5 The quality assessment of Cross-sectional studies**

| **Study** | **Year** | **Q1** | **Q2** | **Q3** | **Q4** | **Q5** | **Q6** | **Q7** | **Q8** | **Q9** | **Q10** | **Q11** | **Total** |
| --- | --- | --- | --- | --- | --- | --- | --- | --- | --- | --- | --- | --- | --- |
| Cursiefen et al. | 2000 | 1 | 1 | 0 | 0 | 0 | 1 | 0 | 1 | 0 | 0 | 0 | 4 |
| Usui et al. | 1991 | 1 | 1 | 0 | 1 | 0 | 1 | 0 | 0 | 0 | 1 | 0 | 5 |

The AHRQ scale was used to evaluate the quality of the Cross-sectional study

**Supplementary Tables 6 Meta-Regression of Heterogeneity Factors in Migraine and glaucoma**

| **Variables** | **Regression Coefficient (95% confidence interval)** | **Stand Error** | **P value** |
| --- | --- | --- | --- |
| Region | 0.11(-0.02-0.23) | 0.06 | 0.10 |
| Study design | 0.03(-0.10-0.17) | 0.06 | 0.61 |
| Data source type | -0.47(-0.75-(-0.20)) | 0.13 | 0.002 |
| Diagnosis of migraine | -0.65(-1.01-(-0.29)) | 0.17 | 0.002 |
| Effect value type | 0.46(0.01-0.90) | 0.21 | 0.045 |
| Female proportion | -0.05(-0.15-0.05) | 0.05 | 0.28 |

**Supplementary Tables 7 Original data extraction and transformation**

| name | years | Effect value type | Effect vaule | LL | DL |  |  |  |  |
| --- | --- | --- | --- | --- | --- | --- | --- | --- | --- |
| Lee et al. | 2026 | adjusted HR | 2.19 | 1.37 | 2.8 | adjusted OR | 2.74 | 1.47 | 3.96 |
| Bar et al. | 2025 | adjusted RR | 1.31 | 1.15 | 1.48 | adjusted OR | 1.32 | 1.15 | 1.5 |
| Kim et al. | 2025 | adjusted HR | 1.238 | 1.16 | 1.321 | adjusted OR | 1.24 | 1.16 | 1.33 |
| Ohn et al. | 2023 | adjusted HR | 1.188 | 1.14 | 1.239 | adjusted OR | 1.19 | 1.14 | 1.24 |
| Funk et al. | 2022 | crude OR | 2.12 | 1.11 | 4.04 | crude OR | 2.12 | 1.11 | 4.04 |
| Jehn-Yu et al. | 2019 | adjusted HR | 1.2 | 0.99 | 1.47 | adjusted OR | 1.2 | 0.99 | 1.47 |
| chen et al. | 2016 | adjusted HR | 1.15 | 0.93 | 1.42 | adjusted OR | 1.15 | 0.93 | 1.42 |
| Furlanetto et al. | 2014 | adjusted HR | 5.737 | 1.46 | 22.727 | adjusted OR | 6.88 | 1.49 | 56.81 |
| Lin et al. | 2010 | adjusted OR | 1.21 | 1.11 | 1.32 | adjusted OR | 1.21 | 1.11 | 1.32 |
| Lin et al. | 2010 | crude OR | 1.26 | 0.54 | 2.95 | crude OR | 1.26 | 0.54 | 2.95 |
| Welinder et al. | 2009 | adjusted OR | 1.32 | 1.13 | 1.54 | adjusted OR | 1.32 | 1.13 | 1.54 |
| Motsko et al. | 2008 | adjusted OR | 1.32 | 1.02 | 1.7 | adjusted OR | 1.32 | 1.02 | 1.7 |
| Girkin et al. | 2004 | crude OR | 4.02 | 1.26 | 12.85 | crude OR | 4.02 | 1.26 | 12.85 |
| Landers et al. | 2002 | adjusted OR | 1 | 0.6 | 1.6 | adjusted OR | 1 | 0.6 | 1.6 |
| Drance et al. | 2001 | adjusted RR | 2.58 | 1.32 | 5.07 | adjusted OR | 3.51 | 1.4 | 11.73 |
| Cursiefen et al. | 2000 | crude OR | 1.42 | 0.63 | 3.22 | crude OR | 1.42 | 0.63 | 3.22 |
| Usui et al. | 1991 | crude OR | 1.52 | 0.3 | 7.78 | crude OR | 1.52 | 0.3 | 7.78 |
| Phelps and Corbett | 1985 | crude OR | 2.16 | 1.01 | 4.6 | crude OR | 2.16 | 1.01 | 4.6 |
| Morgan and Drance | 1975 | crude OR | 2.5 | 0.78 | 8.06 | crude OR | 2.5 | 0.78 | 8.06 |

**Supplementary Figure 1 Sensitivity analysis of the association between migraine and glaucoma**
